# Are smartphones a tool to cope with the fear of childbirth? The correlation between the fear of loss of connection and the fear of childbirth

**DOI:** 10.1590/1806-9282.20240550

**Published:** 2024-08-30

**Authors:** Fadime Bayri Bingol, Arzu Aydoğan, Zeynep Dilşah Karaçam, Derya Çayiroğlu, Büşra Karanfil, Hatice Nur Kaya

**Affiliations:** 1 Marmara University, Faculty of Health Sciences, Department of Midwifery – İstanbul, Türkiye.; 2 Yüksek İhtisas University, Faculty of Health Sciences, Department of Nursing – Ankara, Türkiye.

**Keywords:** Phobic disorders, Smartphone, Fear, Childbirth, Women

## Abstract

**OBJECTIVE:**

With the spread of smartphones, they have become an indispensable part of life, and nomophobia (No-Mobile-Phone Phobia) has emerged.

**METHODS:**

The present research is a cross-sectional study and was conducted with 3,870 primiparous pregnant women between April and May 2022. The research data were collected using the Personal Information Form, Nomophobia Questionnaire, and Wijma Delivery Expectancy/Experience Questionnaire.

**RESULTS:**

The Wijma Delivery Expectancy/Experience Questionnaire score of the pregnant women who participated in the study was 22.3% (n=863) had a clinical fear of childbirth and 19.5% (n=753) had extreme nomophobia. Considering the correlation of the Nomophobia Questionnaire and Wijma Delivery Expectancy/Experience Questionnaire scores with other variables, it was found that the Wijma Delivery Expectancy/Experience Questionnaire scores increased with the increasing Nomophobia Questionnaire total score (p=0.000, r=236) and the Nomophobia Questionnaire total score and fear of childbirth increased with an increase in the daily phone usage time. It was also revealed that women who had smartphone applications related to fetal development had higher nomophobia levels (p=0.0001), while they had a lower fear of childbirth.

**CONCLUSION:**

This study found that one in every five pregnant women was extremely nomophobic and had a clinical fear of childbirth and that nomophobia and the fear of childbirth were correlated at the clinical level. In this regard, women should prefer face-to-face communication rather than smartphones throughout the pregnancy period.

## INTRODUCTION

Smartphones are the main element of communication in modern life and are also one of the indispensable tools as a social accessory regarded as necessary for individuals^1^. Smartphones offer users the convenience of communicating, shopping, accessing social networks or health applications, and accessing unlimited information at any time and place. This causes a device with Internet features, which is developing exponentially every day, to become an integral part of our lives^1^.

Nomophobia (No-Mobile-Phone Phobia) has emerged with the widespread use of smartphones. Nomophobia is a type of phobia that has emerged in the digital age and become widespread after the integration of smartphones into society. Nomophobia is defined as a problem that emerges from the feeling of fear, anxiety, or discomfort due to not having a mobile phone or not being able to access any mobile phone at a certain time. In other words, nomophobia is the fear of feeling incomplete and disconnected from the digital world^2^.

It is known that mobile applications accessible via smartphones are commonly used during pregnancy^3^. Mobile applications used during pregnancy offer a unique window of opportunity for women to obtain information about their health status and change their lifestyles^4^. In this way, women can easily obtain information about being aware of fetal movements, monitoring fetal growth, being aware of changes in themselves, and breastfeeding support^5^. Although it is known that women usually use smartphones during pregnancy for communication, data collection, and education purposes, it is controversial whether the use of smartphones during pregnancy is functional or problematic^4,6^.

Although research on the harm of prolonged screen viewing time to individuals has increased^7^, pregnant women have increasingly turned to mobile health applications instead of face-to-face health services to obtain health information and receive support in recent years^8^. A cohort study examining infants born to 1,378 women between 2014 and 2017 found that the mean smartphone usage time during pregnancy was 29.8 min per day and the fetal growth of infants of women who made mobile phone calls for more than 30 min per day was adversely affected^9^. Furthermore, upon reviewing other studies, it was found that the use of smartphones during pregnancy increased infants’ neurobiological disorders by affecting the immune activation of women^10^, and low birth weight increased with prolonged screen time and rates of infants’ transfer to emergency neonatal intensive care were high^11^. Despite all these risks, the use of smartphones is very common among pregnant women.

It is known that women use mobile applications to obtain information about pregnancy and childbirth processes^12^. Although childbirth is a physiological process, it also causes fear in some women. In this respect, although the main purpose of smartphone use by women is to communicate, this study was conducted considering that the use of smartphones may increase as childbirth approaches to cope with the increasing fear of childbirth and that the increasing fear of childbirth and nomophobia may trigger each other. No study conducted with a pregnant sample and examining the relationship between the fear of childbirth and nomophobia has been found in the literature. In line with this information, the current research was conducted to examine the correlation between the fear of childbirth and nomophobia in primiparous pregnant women.

## METHODS

### Design and location of the study

The present research is a cross-sectional study. It was conducted in Türkiye using an online survey distributed through midwives via WhatsApp groups consisting of pregnant women.

### Sample size and participants

It was determined that the required sample size was 2146 according to the one-tailed independent sample t-test at 99% confidence (1–α), 99% test power (1–β), and d=0.1 effect size using the program G*Power. A total of 4,228 primiparous pregnant women, who were aged between 18 and 45 years, and who voluntarily agreed to take part in the research participated in the study and answered all questions. A total of 358 pregnant women who did not meet the research criteria were excluded from the study [aged 45 years and above (n=18), having fewer than 8 years of education (n=22), not having a spouse with them (n=169), having three or more miscarriages (n=55), having used a smartphone for 5 years or less (n=84), and scoring 138 and more on the Wijma total (n=10)]. The answers given by 3,870 pregnant women were taken into consideration in the data evaluation. The study was conducted in Istanbul, where approximately 20% of Türkiye’s population lives. No sample selection was made in the study, and all pregnant women who agreed to participate in the study between April and May 2022 were included in the study.

### Data collection

The study data were collected through Google Forms. The link to the form was sent via the WhatsApp application, and women were first asked to read the informed consent form. Women who agreed to participate in the study in the informed consent form were included in the study. Midwives sent the link of the study to pregnant women who applied to the health center for routine pregnancy care and follow-up. The researchers’ contact information was added to the survey form, allowing women to contact them in case they encountered any problems. Especially women with a very high fear of childbirth and nomophobia contacted the researchers. In this case, women were directed to receive help from the necessary units.

### Measurement tools

Personal information form: The personal information form created by the researchers was grouped into two sections: demographic characteristics and obstetric history-related characteristics.

Nomophobia Questionnaire (NMP-Q): The scale comprises 20 seven-point Likert-type questions. The study participants can receive a total score varying between 20 and 140. The participant’s score of 20 points indicates that he/she does not have nomophobia, a score between 21 and 59 points indicates a low level of nomophobia, a score between 60 and 99 points indicates a moderate level of nomophobia, and a score between 100 and 140 indicates a high level of nomophobia^13^. In this study, Cronbach’s alpha coefficient of the scale was found to be 0.94.

Wijma Delivery Expectancy/Experience Questionnaire (W-DEQ-A): It is a 33-item Likert-type scale. The study participants can receive a total score varying between 0 and 165. A high item-total score indicates a high level of fear. If the score obtained from the scale is less than 37, it indicates a mild fear of childbirth; if the score obtained from the scale is between 38 and 65, it indicates a moderate fear of childbirth; if the score obtained from the scale is between 66 and 84, it indicates a severe fear of childbirth; if the score obtained from the scale is 85 and above, it indicates a clinical fear of childbirth^14^. In this study, Cronbach’s alpha coefficient of the scale was found to be 0.94.

### Data analysis

The data were analyzed using IBM SPSS Statistics for Windows, Version 25.0. A total of 4,228 pregnant women participated in the study. Continuous variables were defined by mean±standard deviation and median, and categorical variables were defined by number and percentage. For independent group comparisons, the independent sample t-test and one-way analysis of variance (post hoc: Tukey’s test) were performed when parametric test assumptions were met. When parametric test assumptions were not provided, the Mann-Whitney U test and Kruskal-Wallis variance analysis (post hoc: Mann-Whitney U test with the Bonferroni correction) were used for continuous variables. The chi-square test was conducted for categorical variables. Spearman’s correlation coefficient was used for correlations between continuous variables. Logistic regression analysis was used to determine the risk factors for “the status of experiencing a severe fear of childbirth.” Statistical significance was determined as p<0.05.

Ethical considerations: The present research was approved by the Non-Interventional Clinical Research Ethics Committee of Marmara University, Faculty of Health Sciences (No: 2022/07). The ethical principles of the Declaration of Helsinki were adhered to throughout the study, including providing a detailed explanation of the research and maintaining privacy and confidentiality.

## RESULTS

The mean age of the women included in the study was 29.64±3.87 years, the mean gestational week was 23.79±10.55, and the mean education year was 14.89±2.08. It was revealed that 85.9% of the pregnant women had been using smartphones for 10 years or more, and their daily time spent on the internet and social media on smartphones was 207±106.34 min. The number of people that pregnant women constantly followed on social media regarding pregnancy and fetal development was 3.55±1.66, 91.4% downloaded at least one application related to fetal development and monitored fetal development, and the mean number of the applications downloaded with regard to fetal development was 1.63±1.05.

Upon examining the comparative findings, it was found that the NMP-Q scores of pregnant women working in a regular job were significantly higher compared to pregnant women who did not work in a regular job (p=0.001), but there was no statistically significant difference between their fear of childbirth (p˃0.05). Pregnant women with low income had higher nomophobia (p=0.0001) and a higher fear of childbirth (p=0.0001). Likewise, pregnant women with a previous history of consulting a psychotherapist had higher nomophobia (p=0.001) and a higher fear of childbirth (p<0.0001). Table 1 compares the other characteristics and scale scores of pregnant women.

**Table 1:** Comparison of pregnant women’s scale scores according to their demographic and obstetric characteristics (n=3870).

		% (n)	NMP-Q			W-DEQ-A		
			Mean ± SD	Med (IQR)	p	Mean ± SD	Med (IQR)	p
Employment status	Employed	50.3 (n=1947)	75.4 ± 28.33	74 (53–95)	0.001 (z=-3.278)	66.83 ± 23.27	67 (50–83)	0.985 (z=-0.019)
	Unemployed	49.7 (n=1923)	72.38 ± 27.35	71 (51–91)		66.91 ± 22.93	68 (50–83)	
Economic condition	Income less than expenses	23.5 (n=910)	77.32 ± 29.2	76 (55–98)	0.0001 (F=9.757)ab	70.83 ± 23.38	72 (53.75–86)	0.0001 (kw=44.314)abc
	Income equal to expenses	56.4 (n=2181)	72.47 ± 27.42	71 (50.5–92)		66.35 ± 22.55	67 (50–82)	
	Income more than expenses	20.1 (n=779)	73.88 ± 27.31	72 (53–92)		63.68 ± 23.67	63 (46–80)	
Consulting a psychotherapist	Yes	15.2 (n=589)	77.49 ± 28.74	76 (55–97)	0.001 (t=3.401)	71.15 ± 25.45	73 (51.5–87)	0.0001 (z=-4.262)
	No	84.8 (n=3281)	73.25 ± 27.69	72 (51–93)		66.1 ± 22.57	67 (50–82)	
Pregnancy status	Planned pregnancy	80.2 (n=3104)	74.34 ± 27.89	73 (53–94)	0.046 (t=1.999)	66.16 ± 22.87	66.5 (49–83)	0.0001 (z=-3.81)
	Unplanned pregnancy	19.8 (n=766)	72.09 ± 27.84	71 (49–91)		69.73 ± 23.79	72 (53–85)	
Type of conception	Natural pregnancy	92.2 (n=3570)	73.39 ± 27.74	72 (51–93)	0.0001 (t=-3.939)	66.89 ± 23.13	68 (50–83)	0.945 (z=-0.069)
	Pregnancy through treatment	7.8 (n=300)	79.98 ± 28.95	77.5 (57–102.75)		66.64 ± 22.74	67 (51.25–83)	
Health problems during pregnancy	There is a problem	26.1 (n=1009)	75.99 ± 28.85	74 (53–97)	0.007 (t=2.707)	70.65 ± 23.77	72 (54–85)	0.0001 (z=-5.714)
	There is no problem	73.9 (n=2861)	73.16 ± 27.51	72 (51–92)		65.54 ± 22.71	66 (49–82)	
The status of downloading an application with regard to fetal development	Yes, I downloaded	91.4 (n=3538)	74.42 ± 27.72	73 (53–94)	0.0001 (z=-3.949)	66.58 ± 22.95	67 (50–83)	0.017 (z=-2.393)
	No, I did not download	8.6 (n=332)	68.35 ± 29.1	66.5 (44.25–87)		69.93 ± 24.46	70.5 (53–85)	

a: Significant difference between income less than expenses and income equal to expenses. b: Significant difference between income less than expenses and income more than expenses. c: Significant difference between income equal to expenses and income more than expenses.

It was found that the mean W-DEQ-A score of the pregnant women included in the study was 67.06±23.38 and 22.3% had a clinical fear of childbirth. The mean NMP-Q score was 73.89±27.89, and 19.5% had extreme nomophobia. Table 2 compares the fear of childbirth and nomophobia levels of the pregnant women included in the study.

**Table 2:** Comparison of the nomophobia and fear of childbirth total scores of pregnant women (n=3870).

		W-DEQ-A				p
		Mild n (%)	Moderate n (%)	Severe n (%)	Clinical n (%)	
NMP-Q	None	5 (1.1)	5 (0.4)	2 (0.2)	1 (0.1)	0.0001 (cs=196.253)
	Mild	211 (45.9)	530 (38.7)	370 (31.4)	185 (21.4)	
	Moderate	179 (38.9)	649 (47.4)	589 (50)	391 (45.3)	
	Extreme	65 (14.1)	184 (13.5)	218 (18.5)	286 (33.1)	

When the relationship between the NMP-Q and W-DEQ-A scores of the pregnant women included in the study and other variables was examined by correlation analysis, it was found that the W-DEQ-A total score increased with the increasing NMP-Q total score (p=0.000, r=236), the NMP-Q total score increased with the increased number of years of telephone use (p=0.000, r=0.058), the NMP-Q total score (p=0.000, r=0.256), and fear of childbirth increased (p=0.000, r=0.087) with the increase in the daily phone usage time, and the NMP-Q total score increased (p=0.000, r=0.086), by the W-DEQ-A total score decreased (p=0.006, r=-0.044) with the increasing number of people followed on social media with regard to pregnancy and childbirth.

The risk factors affecting the status of experiencing a severe fear of childbirth were examined with both univariate and multivariate models. Considering univariate results, it was revealed that having a low income, increased number of curettages, having an unplanned pregnancy, experiencing health problems during pregnancy, having previously consulted a psychotherapist, increased daily phone usage time, not having received online education, and an increase in all subscales, particularly the nomophobia total score, increased the risk of experiencing a severe fear of childbirth (p<0.05). The multivariate model established with the risk factors found to be significant in univariate terms showed that having a low income, having an unplanned pregnancy, experiencing health problems during pregnancy, not having received online education, and an increase in the total nomophobia score were among the risk factors that statistically significantly increased the risk of having a severe fear of childbirth (p<0.05) (Table 3).

**Table 3:** Risk factors affecting the status of experiencing a severe fear of childbirth.

		Univariate			Multiple		
		p	OR	95%CI for OR	p	OR	95%CI for OR
Ref: low income	Economic (equal)	0.0001	0.676	0.566–0.806	0.004	0.762	0.633–0.916
	Economic (rich)	0.0001	0.56	0.444–0.707	0.0001	0.628	0.493–0.799
–	Curettage	0.019	1.204	1.03–1.406	0.170	1.122	0.952–1.323
Ref: planned	Pregnancy plan (unplanned)	0.011	1.267	1.055–1.522	0.008	1.293	1.068–1.566
Ref: no	Problem during pregnancy (yes)	0.0001	1.49	1.263–1.758	0.0001	1.370	1.152–1.629
Ref: no	Previous psychiatric diagnosis (yes)	0.0001	1.674	1.378–2.034	0.177	1.265	0.899–1.78
Ref: no	Previous psychiatric drug (evet)	0.0001	1.586	1.33–1.891	0.191	1.229	0.902–1.673
–	Daily phone time code	0.0001	1.102	1.056–1.149	0.714	1.009	0.964–1.055
Ref: no	Online education (yes)	0.006	0.589	0.404–0.86	0.013	0.609	0.413–0.9
–	Nomototal	0.0001	1.019	1.017–1.022	0.0001	1.019	1.016–1.022
Ref: not advanced	Nomophobic (advanced)	0.0001	2.696	2.268–3.204	–	–	–

OR: odds ratio; 95%CI: 95% confidence interval; logistic regression analysis.

## DISCUSSION

Smartphones, the biggest nondrug addiction of the 21st century, isolate individuals from the real world by making them addicted to the virtual world. Similar to this study, a study conducted with pregnant women in China showed that women use TV, computers, and smartphones for long periods and this use is common^15^. Moreover, it was found that young pregnant women with low education levels in Japan used mobile phones excessively^16^.

It is controversial whether using smartphones and following health-related applications during pregnancy is beneficial. A systematic review showed that applications did not continuously provide useful or accurate nutritional information^6^, and smartphone applications usually were of low to medium quality^17^. All of th e middle 10 pregnancy applications in Australia, selected using download numbers and star ratings, were determined to be inadequate in terms of quality, practicality, and functionality, and issues related to the accuracy of the information in the applications, privacy, and security should also be addressed, and the need for more evidence to reveal whether these applications affect pregnancy outcomes is also stressed^18^.

A randomized controlled trial found that “HealthyMoms” had the potential to promote healthy eating behaviors and reduce weight gain during pregnancy in overweight and obese women but had no impact on other women^19^. It is also reported that applications for health behaviors of pregnant women, such as monitoring fetal movements, maintaining a healthy weight during pregnancy, and breastfeeding, may be useful for providing health information and communication in low- and middle-income countries^20^. A review investigating the use of smartphone applications during pregnancy showed useful and acceptable tools for clinicians^21^. This study determined that one in every five pregnant women was extremely nomophobic. A systematic review study determined that women and young individuals suffer from nomophobia more^22^. While smartphone applications are increasingly used every day, the WHO^23^ is conducting studies such as the “Classification of Digital Health Interventions.”

## CONCLUSION

This study found that one in every five pregnant women was extremely nomophobic and had a clinical fear of birth, that nomophobia and fear of childbirth were interrelated at the clinical level, and that both nomophobia and fear of childbirth scores increased with the increasing duration of daily phone use. The power of technological progress cannot be ignored. However, women should be careful about possible harms during pregnancy, and the use of smartphones should be limited as much as possible. Pregnant women should be supported in spending time in the real world rather than the virtual world, and human-to-human interactions and face-to-face connections should be established at an optimum level. Health professionals should provide adequate and qualified face-to-face care to pregnant women.

## ETHICAL APPROVAL

The study was approved by the Marmara University Ethical Committee of Health Sciences Faculty (2022/07).
